# Building climate-sensitive nutrition programmes

**DOI:** 10.2471/BLT.21.285589

**Published:** 2021-12-02

**Authors:** Hannah Nissan, Will Simmons, Shauna M Downs

**Affiliations:** a Grantham Institute for Climate Change and the Environment, London School of Economics and Political Science, London, England.; b Department of Epidemiology, Columbia University Mailman School of Public Health, New York, United States of America (USA).; c Department of Urban–Global Public Health, Rutgers School of Public Health, One Riverfront Plaza, Suite 1020, Newark, New Jersey, NJ 07102, USA.

## Abstract

The food system and climate are closely interconnected. Although most research has focused on the need to adopt a plant-based diet to help mitigate climate change, there is also an urgent need to examine the effects of climate change on food systems to adapt to climate change. A systems approach can help identify the pathways through which climate influences food systems, thereby ensuring that programmes combating malnutrition take climate into account. Although little is known about how climate considerations are currently incorporated into nutrition programming, climate information services have the potential to help target the delivery of interventions for at-risk populations and reduce climate-related disruption during their implementation. To ensure climate services provide timely information relevant to nutrition programmes, it is important to fill gaps in our knowledge about the influence of climate variability on food supply chains. A proposed roadmap for developing climate-sensitive nutrition programmes recommends: (i) research aimed at achieving a better understanding of the pathways through which climate influences diet and nutrition, including any time lags; (ii) the identification of entry points for climate information into the decision-making process for nutrition programme delivery; and (iii) capacity-building and training programmes to better equip public health practitioners with the knowledge, confidence and motivation to incorporate climate resilience into nutrition programmes. With sustained investment in capacity-building, data collection and analysis, climate information services can be developed to provide the data, analyses and forecasts needed to ensure nutrition programmes target their interventions where and when they are most needed.

## Résumé

Les systèmes alimentaire et climatique sont étroitement liés. Bien que la plupart des recherches se concentrent sur le besoin d'adopter un régime végétarien pour contribuer à atténuer le changement climatique, il est également urgent d'examiner les effets de ce changement climatique sur les systèmes alimentaires afin de les adapter en conséquence. Une approche systémique peut aider à déterminer dans quelle mesure le climat influence les systèmes alimentaires, et dès lors à faire en sorte que les programmes de lutte contre la malnutrition en tiennent compte. On ignore encore sous quelle forme les considérations climatiques sont actuellement intégrées dans les programmes de nutrition. Cependant, les services climatologiques peuvent contribuer à cibler le déploiement d'interventions pour les populations à risque, ainsi qu'à réduire les perturbations causées par le climat au cours de leur mise en œuvre. Pour veiller à ce que ces services fournissent à point nommé des informations utiles aux programmes de nutrition, il faut impérativement combler le manque de connaissances en matière d'impact des variations climatiques sur les chaînes d'approvisionnement alimentaire. La feuille de route proposée pour l'élaboration de programmes de nutrition adaptés au changement climatique recommande: (i) de mener des recherches visant à mieux comprendre dans quelle mesure le climat influence l'alimentation et la nutrition, en tenant compte des éventuels décalages temporelshoraires; (ii) d'identifier les points d'entrée des informations relatives au climat dans le processus décisionnel de déploiement des programmes alimentaires; et enfin, (iii) de développer des plans de formation et de renforcement des capacités afin que les professionnels de la santé publique disposent des connaissances, de la confiance et de la motivation nécessaires pour intégrer l'adaptation au changement climatique dans les programmes de nutrition. En investissant durablement dans le renforcement des capacités ainsi que dans la collecte et l'analyse de données, il est possible d'instaurer des services climatologiques qui communiqueront les informations, analyses et prévisions requises pour que les programmes de nutrition organisent leurs actions à l'endroit et au moment où elles deviennent indispensables.

## Resumen

El sistema alimentario y el clima están muy interconectados. Aunque la mayoría de las investigaciones se han centrado en la necesidad de adoptar una dieta basada en el consumo de plantas para ayudar a mitigar el cambio climático, también es urgente analizar los efectos del cambio climático en los sistemas alimentarios para adaptarse al mismo. Un enfoque sistémico puede ayudar a identificar las vías a través de las que el clima influye en los sistemas alimentarios, garantizando así que los programas de lucha contra la malnutrición tengan en cuenta el clima. Si bien se sabe poco sobre cómo se incorporan en la actualidad los aspectos climáticos a los programas de nutrición, los servicios de información climática tienen el potencial de ayudar a orientar las intervenciones hacia las poblaciones de riesgo y a reducir los trastornos relacionados con el clima durante su aplicación. Para garantizar que los servicios climáticos proporcionen información oportuna y relevante a los programas de nutrición, es importante solucionar las carencias de nuestros conocimientos sobre la influencia de la variabilidad climática en las cadenas de suministro de alimentos. Una hoja de ruta propuesta para desarrollar programas de nutrición sensibles al clima recomienda i) la investigación destinada a lograr una mejor comprensión de las vías a través de las que el clima influye en la dieta y en la nutrición, incluidos los retrasos; ii) la identificación de los puntos de entrada de la información sobre el clima en el proceso de toma de decisiones para la ejecución de los programas de nutrición; y iii) los programas de creación de capacidad y formación para preparar mejor a los profesionales de la salud pública con los conocimientos, la confianza y la motivación que permitan incorporar la adaptación al clima en los programas de nutrición. Si se invierte de manera sostenida en la creación de capacidades, la recopilación y el análisis de datos, se pueden desarrollar servicios de información climática que proporcionen los datos, los análisis y las previsiones necesarios para garantizar que los programas de nutrición orienten sus intervenciones donde y cuando más se necesiten.

## ملخص

هناك ترابط وثيق بين نظام الغذاء والمناخ. على الرغم من أن معظم الأبحاث قد ركزت على الحاجة إلى اتباع نظام غذائي نباتي للمساعدة في التخفيف من تغير المناخ، إلا أن هناك أيضًا حاجة ملحة لدراسة آثار تغير المناخ على النظم الغذائية للتكيف مع تغير المناخ. يمكن أن يساعد أسلوب النظم في تحديد المسارات التي من خلالها يؤثر المناخ على النظم الغذائية، وبالتالي يضمن أن تأخذ برامج مكافحة سوء التغذية، المناخ في الاعتبار. على الرغم من أنه لا يُعرف سوى القليل عن كيفية دمج الاعتبارات المناخية حاليًا في برمجة التغذية، إلا أن خدمات المعلومات المناخية لديها القدرة على المساعدة في استهداف تقديم التدخلات للسكان المعرضين للخطر، والحد من الاضطرابات المرتبطة بالمناخ أثناء تنفيذها. لضمان أن الخدمات المناخية توفر المعلومات ذات الصلة ببرامج التغذية في الوقت المناسب، فإنه من المهم سد الفجوات في معرفتنا بخصوص تأثير تقلب المناخ على سلاسل الإمداد الغذائي. توصي خارطة طريق مقترحة لتطوير برامج التغذية المعتمدة على المناخ بما يلي: (1) البحث الذي يهدف إلى تحقيق فهم أفضل للمسارات التي يؤثر المناخ من خلالها على النظام الغذائي والتغذية، بما في ذلك أي فترات تأخير؛ و(2) تحديد نقاط دخول معلومات المناخ في عملية صنع القرار لتقديم برنامج التغذية؛ و(3) بناء القدرات وبرامج التدريب لتزويد ممارسي الصحة العامة بالمعرفة والثقة والتحفيز، بشكل أفضل، لإدماج المرونة المناخية في برامج التغذية. من خلال الاستثمار المستدام في بناء القدرات وجمع البيانات وتحليلها، يمكن تطوير خدمات المعلومات المناخية لتوفير البيانات، والتحليلات، والتنبؤات اللازمة لضمان أن تستهدف برامج التغذية تدخلاتها حيثما وأينما تكون الحاجة إليها في أقصى درجاتها.

## 摘要

粮食体系和气候密不可分。虽然大部分研究都集中在采取以植物为主的饮食来帮助缓解气候变化，但我们意识到，当前还迫切需要检测气候变化对粮食体系的影响来适应气候的变化。系统方法有助于识别气候对粮食体系的影响路径，因此在制定解决营养问题的计划时应该考虑气候因素。尽管目前对如何将气候因素纳入营养计划尚不明确，但气候信息服务可能有助于针对性地为风险群体提供干预措施，并降低实施过程中与气候有关的破坏。为了确保气候服务及时提供与营养计划相关的信息，有必要填补我们在气候变化对粮食供应链的影响方面的知识空白。针对拟议的气候敏感型营养计划的制定路线图，建议如下：(i) 开展研究，目的在于更好地理解气候对饮食和营养的影响途径，包括任何时滞效应；(ii) 确定气候信息纳入营养计划决策流程的切入点；以及 (iii) 开展能力培养和培训计划，提高公共卫生从业人员的素质，让他们具备知识、自信心和动力来将气候适应性融入营养计划。通过在能力培养、数据收集和分析方面的持续性投资，可以发展气候信息服务以提供所需数据、分析和预测，确保在最需要的时间和地点有针对性地将营养计划纳入干预措施。

## Резюме

Продовольственная система и климат тесно взаимосвязаны. Несмотря на то что большинство исследований сосредоточены на необходимости перехода на растительную диету для смягчения последствий изменения климата, существует также острая необходимость в изучении воздействия последствий климатических изменений на продовольственные системы с целью адаптации к изменениям климата. Системный подход может способствовать выявлению путей воздействия климатических условий на продовольственные системы, тем самым обеспечивая разработку программ борьбы с нарушением питания с учетом климата. Хотя мало что известно о том, как в настоящее время осуществляется учет влияния климата в процессе разработки программ в области питания, службы информации по вопросам изменения климата могут помочь в реализации соответствующих мер для групп населения, подверженных риску, а также для смягчения последствий, связанных с изменениями климата, при реализации таких программ. Чтобы службы информации по вопросам изменения климата предоставляли своевременную информацию, необходимую для разработки программ питания, важно устранить пробелы в наших познаниях о влиянии изменчивости климата на цепочки поставок продуктов питания. Предлагаемая дорожная карта для разработки программ питания с учетом климатических условий рекомендует: (i) проведение исследований, направленных на углубление понимания путей воздействия климата на диету и питание, включая любые задержки во времени; (ii) определение отправных точек для включения информации о климате в процесс принятия решений по реализации программы питания; (iii) осуществление программы по наращиванию потенциала и подготовке кадров, чтобы обеспечить специалистов в области общественного здравоохранения знаниями, уверенностью и мотивацией для включения вопросов обеспечения устойчивости к изменению климата в программы питания. При устойчивом вложении сил и средств в программы наращивания потенциала, сбор и анализ данных можно разработать службы информации по вопросам изменения климата для предоставления данных, аналитических отчетов и прогнозов, которые необходимы для того, чтобы реализация мер в рамках программ питания осуществлялась в нужном месте и в нужное время.

## Introduction

The food system and climate are closely interconnected. To date, most research has focused on climate change – specifically on the environmental footprint of our diets and on how we could reduce that footprint by shifting towards a plant-based diet.^1^^–^^4^ Food systems clearly affect the climate: together food production and food supply chains account for approximately one third of all greenhouse gas emissions, 70% of fresh water consumption and a substantial loss of biodiversity.^4^^–^^7^ However, the relationship is not one-way. Seasonality, climate variability (including extreme events) and climate change can disrupt food production and have a broad effect on the food system and the people and organizations within it.^8^^,^^9^ In fact, climate change is already underway and further warming is unavoidable, even if we meet the most ambitious emissions targets.^10^^,^^11^ Thus, although changing our diet will be critical for mitigating climate change, it is also important to examine the interconnections between climate and food systems from the perspective of adapting to climate change – and to climate variability on all timescales.

Previous studies of the impact of climate on food systems have largely focused on the production of staple crops.^8^ Less is known about how the effects of climate variability propagate through the food system as a whole.^8^ Research on seasonal variations in climate demonstrate that climate variability can affect the availability and affordability of food,^12^^–^^15^ as well as nutrition outcomes, such as wasting.^16^^,^^17^ There is, however, a need for more studies, especially for comprehensive examinations of the influence of climate variability on food supply chains, on the food environment (i.e. the consumer interface with the food system, encompassing the availability, affordability, convenience and desirability of foods) and on individual factors guiding the purchase and consumption of food. These gaps in knowledge impede our ability to address the growing burden of malnutrition globally, where one in nine people goes hungry and one in three is overweight or obese.^18^ In turn, poor nutrition and inadequate diets threaten progress towards several of the United Nations’ sustainable development goals (SDGs) – including SDG 2, to “End hunger, achieve food security and improved nutrition, and promote sustainable agriculture” – because they affect educational attainment, labour productivity, inequality and other important components of development.^19^

A systems approach is needed to capture the potential magnitude of the influence of the climate on food systems. A food systems approach can describe and analyse the different elements of a food system (e.g. food supply chains and the food environment) and the relationships among these elements.^20^ The approach can examine both activities related to food production, processing and distribution and the effects of these activities on food security, nutrition, society, the economy and the environment.^20^ Adopting such an approach has several benefits, such as identifying: (i) the root causes of specific food system outcomes; and (ii) innovative ways of addressing them across different sectors and timescales.^20^^,^^21^ In addition, this approach could increase our understanding of the multiple, interconnected pathways through which climate can affect diets and nutrition, thereby helping to identify strategies for adapting to climate variability and climate change. More specifically, a food systems approach could enable us to evaluate different adaptative responses to climate change and their potential knock-on effects (including unintended consequences) for other parts of the system, such as diet and nutrition.

**Fig. 1:**
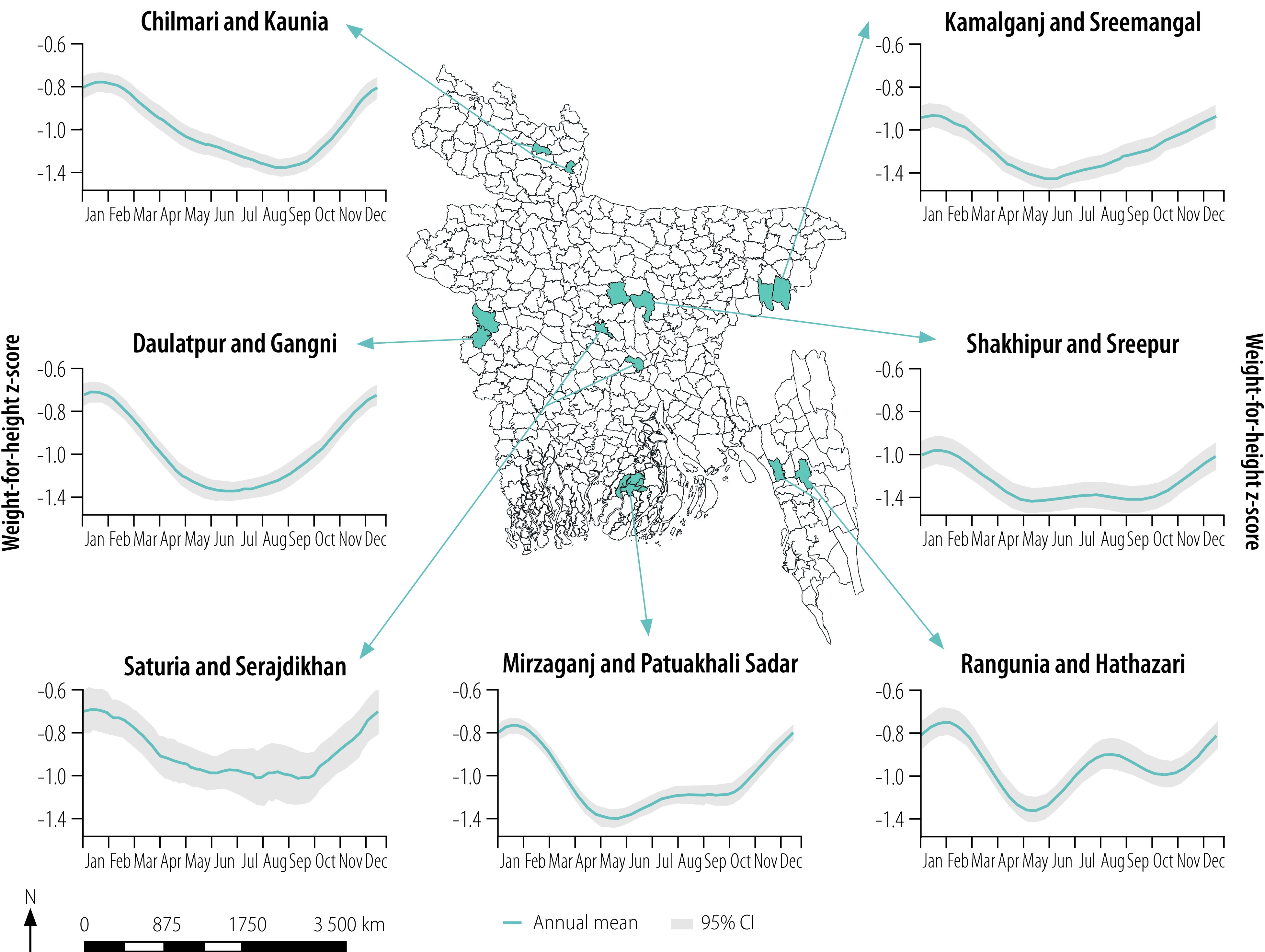
Aggregate annual weight-for-height cycles in children younger than 5 years, Bangladesh, 1990–2006

Many climate shocks and stressors are highly seasonal and their influence on food systems and health depends on when they occur during the year. The effect of seasonal climate variations on nutrition outcomes, such as wasting or weight-for-height, can be observed today in many low- and middle-income countries that experience so-called hunger seasons (Box 1 and Fig. 1).^24^^,^^25^ Seasonal patterns influence several food system activities and could also affect nutrition through indirect mediators, such as infectious disease, access to health-care facilities or household income.^24^^–^^26^ For example, a seasonal reduction in income could lead to a decrease in food and health expenditure.^27^^,^^28^ Variations in temperature, humidity and rainfall, as well as extreme events such as floods, droughts and heat waves, can affect the transmission of infectious and vector-borne diseases.^29^^–^^33^ The contamination of crops and animal feed with aflatoxins (i.e. carcinogens produced by certain moulds), caused by drought or heat stress during production or by hot and humid conditions during storage and transportation,^34^ is associated with a range of health effects, including an increased risk of stunting in children younger than 5 years and an increased risk of liver cancer.^35^^,^^36^ For crops at risk of aflatoxin contamination, planting schedules are generally designed to avoid predisposing conditions. However, increased climate variability has made this strategy less effective.^34^ Increased susceptibility to aflatoxins may be exacerbated by climate shocks (e.g. storms, floods and fires) that create physical barriers to accessing health care – an important mediator of nutrition outcomes given the relationship between health and nutritional status.

Box 1 **Seasonal weight-for-height cycles in children younger than 5 years, Bangladesh, 1990–2006**

In Bangladesh, as in many low- and middle-income countries, nutrition outcomes (e.g. weight-for-height) exhibit strong seasonal cycles, for reasons that are only partially understood.^22^ Better characterization of this seasonality and its spatial variation could help nutrition programmes target interventions towards the populations most at risk.

To investigate seasonal undernutrition in Bangladesh, we used data from the Nutrition Surveillance Project – a national programme administered by Helen Keller International that has gathered data since 1990.^23^ This data set is one of the most comprehensive nutrition resources globally: it comprises nationally representative nutrition, socioeconomic and other contextual data at individual and household levels in Bangladesh.

We calculated weight-for-height *z*-scores (a score under −2 indicates wasting and poor nutrition) for children who were younger than 5 years between 1990 and 2006 and plotted multiyear, seasonal, weight-for-height cycles for 22 matched pairs of subdistricts (Fig. 1). Distinct changes in the average weight-for-height were observed across the seasons: the period from November to February was consistently associated with less severe undernutrition, whereas the period from June to September was associated with more severe undernutrition. The magnitude and timing of the weight-for-height cycles varied with both season and geographical location. For example, in the northern subdistricts of Chilmari and Kaunia, the descent into the so-called hunger season was slow and the weight-for-height *z*-score reached its lowest value between September and October. In the south-eastern subdistricts of Rangunia and Hathazari, in contrast, the lowest weight-for-height *z*-score was observed between May and June and there was evidence of a rebound period between June and October. Although the timing and magnitude of the weight-for-height cycles varied across subdistricts, there was some suggestion of broader geographical clustering.

The high temporal resolution of the data from the Bangladesh Nutrition Surveillance Project (now the Food Security and Nutrition Surveillance Project) makes it possible to investigate the relationships between climate and nutrition across time. In contrast, nutrition data based on less frequent sampling (e.g. Demographic Health Survey data) cannot be compared with climate data on a meaningful timescale. However, even the current Bangladesh data do not cover a sufficiently long time period for causal inferences to be drawn: claims of any relationship between climate and nutrition will remain suggestive until repeated assessments of individuals demonstrate a pattern of nutrition change over time.

CI: confidence interval.

Notes: Mean weight-for-height *z*-scores were calculated by comparing each child’s weight-for-height with values derived from sex-specific, child growth curves generated by the World Health Organization in 2006. Weight-for-height *z*-scores were calculated on a daily basis, then smoothed across calendar days 1 to 365 for presentation (with leap days dropped). A low z-score indicates worse wasting. The individual graphs show aggregate annual values for pairs of subdistricts in Bangladesh for the period 1990 to 2006, smoothed using a sixth-order Fourier series. The analysis considered a total of 796 996 daily observations overall. The shaded areas represent 95% CI using 500 bootstrap samples.

## Climate services

Nutrition programmes aim to address the underlying and immediate drivers of malnutrition.^37^ However, little is known about how these programmes can incorporate climate considerations into their operations. Given shifting climate patterns and increasing climate variability, nutrition programmes should be climate-sensitive to ensure they can target interventions towards at-risk populations and reduce climate-related disruptions.

How can we use knowledge about the climate to improve the effectiveness of nutrition programmes? If the critical drivers of undernutrition are known, monitoring these drivers and predicting their evolution could improve nutrition outcomes by facilitating better targeting of interventions. There are precedents for using climate information to support practical decision-making in other areas of public health. In Ethiopia, for example, the availability of user-friendly meteorological data and analyses at the district level has enabled public health practitioners to assess when conditions are suitable for malaria transmission.^38^ When combined with appropriate training, these climate information tools can be used to plan malaria control programmes and focus resources in areas where the potential for disease transmission is high. Similar approaches can be applied to nutrition programming. In Bangladesh, for example, households predicted to experience extreme flooding were given an anticipatory cash transfer.^39^ The money was spent mostly on food and water, which led to higher child and adult food consumption and greater well-being than in households that did not receive the transfer.^39^

The findings of a scoping review in six countries indicate that nutrition programmes do not currently take climate resilience into account, despite widespread recognition that climate could be an important driver of nutrition outcomes.^40^^,^^41^ Initially, interviews with stakeholders suggested that appropriate climate information was either not available or not easy to use. However, subsequent discussions revealed a lack of knowledge about how climate affects nutrition and, consequently, about what actions could be taken to mitigate and plan for the effects of climate variability and change and thus about the type of climate information that would be useful for guiding those actions.

## A roadmap

### Climate–nutrition pathways

The minimum requirement for a useful climate information service is that it provides timely information relevant to the task at hand in an understandable format. Meeting these criteria requires an understanding of the pathways through which climate influences food systems, including any time lags, so that the relevant climate variables can be identified and linked to timely interventions. Given the natural time lags between observed changes in climate and nutrition outcomes,^42^^,^^43^ climate observations could be used to predict these outcomes in a particular population. If the lag is too short to enable sufficient time to intervene, forecasts – if skilful – can provide additional lead time for action to be taken.^44^ Today, forecasts of weather events such as hot or cold spells can be accurate up to about 10 days in advance at most but usually the lead time is much shorter.^45^ Beyond this limit, we can rely on climate forecasts, which predict the slower-moving components of the climate system that are captured by weather statistics over longer periods of time. The climate varies on multiple timescales (or frequencies), including subseasonal variations, the seasonal cycle and longer-term fluctuations from year to year and across decades. Gradual, nonlinear trends linked to anthropogenic climate change are superimposed over this natural variability, which is itself also being affected by changes in the frequency of extreme events and in the timing of the seasons. Seasonal forecasts are currently the most widely available, and skilful,^44^ type of climate forecast.^41^ Most seasonal forecasts indicate whether the coming season is likely to be broadly wetter or drier, or hotter or colder, than the long-term average calculated over several years. Such forecasts may or may not be predictive of nutrition outcomes. Better understanding of those aspects of the weather and climate that are important for nutrition outcomes will result in the expansion of targeted information services that monitor and predict the most relevant variables.

Researchers can increase the value of their research to practitioners by framing it in terms of the practical tools available to act upon the knowledge it generates. Current epidemiological studies on nutritional vulnerabilities seldom differentiate the effects of climate variations occurring on different timescales, such as extreme weather events, seasonal patterns or interannual variability. If the aim is to devise actions that reduce undernutrition by harnessing climate information, we could frame epidemiological research in terms of the timescales on which climate variability occurs. For example, one hypothesis to test might be that particular types of weather shocks, such as flooding or heat waves, result in a subsequent peak in undernutrition. The findings could guide the way surveillance data (given a sufficient lag in the impact of the shock) or weather forecasts (if more lead time is needed to act) are used to inform the delivery of nutrition interventions (e.g. cash transfers). To explore whether seasonal forecasts could help target nutrition programmes to reduce child undernutrition during the hunger season, we might, for example, test the hypothesis that the year-to-year variation in summer rainfall predicts the year-to-year variation in the rate of child wasting during the hunger season (Box 1 and Fig. 1). Recent research found that a warm temperature anomaly in the previous year was associated with lower dietary diversity in the current year among children in several regions.^46^ This finding suggests that seasonal forecasts could potentially be used to guide nutrition interventions. However, further research is needed to characterize the timing and nature of the temperature anomalies and to identify pathways through which dietary diversity is affected. It is unclear, for example, whether temperature extremes or increases in average temperature are responsible for the lack of diversity or whether the season in which an anomaly occurs might be important. If the goal is to inform practical adaptation, then understanding the relevant climate metric and the timing of exposures during the year is critical. Otherwise, how would we know when and how to intervene?

The most important prerequisite for understanding how climate influences food systems is the availability of high-quality climate and nutrition data sets. Investment is required to maintain and upgrade national data sets at the temporal and spatial resolutions required to support local nutrition programmes (e.g. the Enhancing National Climate Services climate data programme).^47^ Developing these data sets will take years and research cannot progress substantially without them. However, the statistical methods currently available for analysing existing data sets (which may have incongruent spatial and temporal resolutions) are generally underused. There is also a strong case for climate-proofing public health data sets such that they can be analysed along with climate data. For example, to understand how nutrition varies with the seasons, data must be collected several times a year. Moreover, these data must be collected every year as part of a sustained data collection programme if we are to anticipate variations in the timing and magnitude of regular peaks in undernutrition from year to year. Although sustained investment in data is needed, it may often be possible to modify existing data collection programmes relatively easily, for example, by ensuring that surveys are conducted at a suitable frequency, at the appropriate times of the year and in locations representative of the regional climate.

### Entry points for information 

**Fig. 2:**
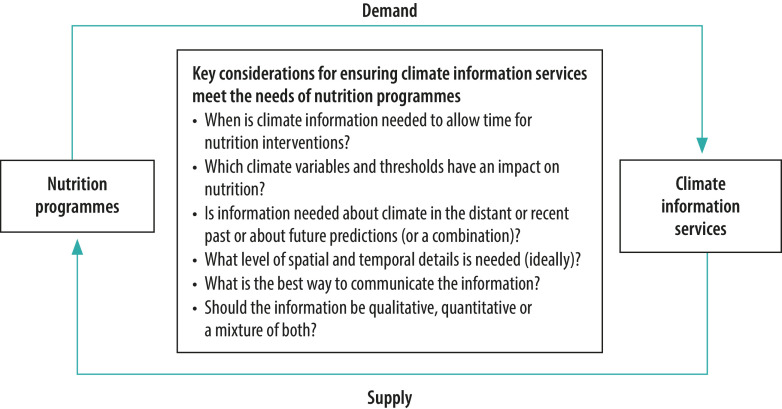
Key questions on how to make climate information services relevant to programmes combating malnutrition

We foresee several ways in which climate data could assist existing or future nutrition programmes. Data could be helpful in: (i) targeting nutrition interventions more effectively; (ii) improving the timing of nutrition programme delivery (particularly of emergency nutrition); and (iii) informing national plans and policies for long-term nutrition planning and preparedness.^43^ Research is needed to identify entry points for climate information into the decision-making process for nutrition programme delivery. Knowledge about the timing of key decisions, about the physical scale of the planned interventions and about how decision-makers would prefer to receive climate information, for example, could help identify these entry points and clarify the nature of the climate information services required. Fig. 2 shows some key questions relevant to aligning the supply and demand of climate information services for nutrition programmes. Sustained engagement between the nutrition and climate information service communities will be vital for ensuring that this process leads to useful outcomes and that climate information services remain relevant.

With an increased understanding of the context in which decisions about nutrition programmes are made, climate information service providers (e.g. national and regional meteorological services) can develop targeted information products to support these decisions. Climate forecasts, data and knowledge can be communicated using a variety of formats or presentations and the level of technical detail can be matched to the intended audience. For example, information could be distilled into simple messages or presented using sophisticated online tools that allow individuals to explore the climate data and forecasts relevant to their area of interest.^48^

### Building capacity and expertise

To achieve practical results, research into how climate information can be incorporated into nutrition programmes must be complemented by a capacity-building programme to ensure that public health practitioners acquire the knowledge, confidence and motivation to make nutrition programmes more climate-resilient. Capacity-building will involve more than a training programme; it should also help develop expertise in the transdisciplinary field of climate, health and food systems. Today, there is a growing recognition that people working in the food system need to undergo interdisciplinary training to better equip them to tackle the complex challenges faced by the food system, including increased climate variability.^49^ However, such cross-cutting approaches are not yet the norm. Similarly, climate information service providers still have some way to go to provide actionable information. Progress will depend on their willingness and capacity to approach the problem of adapting to climate change from the perspective of decision-makers.^50^

Inevitably there will be gaps between the information that climate scientists can reliably provide and the level of precision and lead times desired by decision-makers. This mismatch can be a reason for inaction. Two key components of capacity-building and training programmes – which cannot be provided by meteorologists alone – are: (i) to develop an understanding of the limitations of climate data and predictions; and (ii) to learn how to combine these data with other information and knowledge to support effective local action. We can learn lessons from the history of seasonal forecasting, where international standards were developed by the meteorological community largely in isolation from the stakeholders who stood to benefit. This approach contributed to confusion about how to use seasonal forecasts (or whether to use them at all) despite the World Meteorological Organization holding annual Climate Outlook Forums around the world to increase their usage.^50^

## Conclusions

We propose that the effect of climate on nutrition should be managed using a systems approach that goes beyond simply considering food production. Although the impact of food systems on climate change has already been studied, less attention has been paid to the multiple ways in which climate, in turn, influences food systems or to the pathways through which that influence operates. The specific effects of climate on food systems can be challenging to quantify and predict but they must be considered when planning food and nutrition programmes if the SDGs are to be achieved. To begin, research is urgently needed in three areas: (i) epidemiological research into the pathways through which climate variability and climate shocks influence diet and nutrition and into the timescales over which these influences act; (ii) research to identify and contextualize entry points for incorporating climate information into nutrition programmes to support programme delivery; and (iii) methodological research to develop the conceptual models and analytical tools needed to pull together findings from these different strands of research. Achieving a good understanding of the effects of climate on food systems as a whole involves considering the multiple spatial and temporal scales on which these effects operate and on employing expertise from a range of disciplines. With sustained investment in capacity-building and data, knowledge of the interactions between climate and food systems could be used to develop climate information services that can provide the data, analyses and forecasts needed to ensure nutrition programmes target their interventions where and when they are most needed.
